# Suppressing Endothelial–Mesenchymal Transition Through the Histone Deacetylase 1/GATA Binding Protein 4 Pathway: The Mechanism of Protocatechuic Acid Against Myocardial Fibrosis Revealed by an Integrated Study

**DOI:** 10.3390/biology15020206

**Published:** 2026-01-22

**Authors:** Chengsi Jin, Chongyu Shao, Guanfeng Xu, Haitong Wan

**Affiliations:** 1 College of Basic Medical Sciences, Zhejiang Chinese Medical University, Hangzhou 310053, China; 2 Zhejiang Key Laboratory of Chinese Medicine for Cardiovascular and Cerebrovascular Disease, Hangzhou 310053, China; 3 Academy of Chinese Medical Sciences, Henan University of Chinese Medicine, Zhengzhou 450046, China

**Keywords:** protocatechuic acid, myocardial fibrosis, EndMT, HDAC1/GATA4

## Abstract

Background: Myocardial fibrosis, a central pathological process leading to heart failure, lacks specific mechanism-based therapies. Although the anti-inflammatory activity of the natural compound protocatechuic acid is recognized, its direct anti-fibrotic mechanism, particularly concerning the critical role of endothelial–mesenchymal transition (EndMT), remains unexplored. This study aimed to investigate the protective effects and underlying mechanisms of protocatechuic acid. Methods: The study employed both in vivo and in vitro models. For in vivo evaluation, a rat model of myocardial fibrosis was induced by isoproterenol hydrochloride (ISO). For in vitro analysis, human umbilical vein endothelial cells (HUVECs) were stimulated with angiotensin II (Ang II) and subjected to siRNA-mediated histone deacetylase 1 (HDAC1) knockdown, alongside a co-culture model involving HUVECs and the AC16 human cardiomyocyte cells. Additionally, molecular docking and dynamics simulations were performed to evaluate the binding affinity and stability of protocatechuic acid with the target protein, HDAC1. Results: In vivo, protocatechuic acid significantly improved cardiac function, attenuated pathological injury, and reduced collagen deposition in ISO-induced fibrotic rats. It also potently suppressed inflammatory responses and inhibited the EndMT process. These beneficial effects were associated with decreased HDAC1 and increased GATA binding protein 4 (GATA4) expression in perivascular regions, which suggests the modulation of the HDAC1/GATA4 pathway. In vitro, protocatechuic acid suppressed Ang II-induced endothelial inflammation in HUVECs. This effect was replicated by HDAC1 knockdown, thus confirming that the HDAC1/GATA4 pathway mediates its anti-inflammatory action at the cellular level. Furthermore, molecular docking and dynamics simulations indicated that protocatechuic acid stably binds to a key target, HDAC1. Conclusions: Protocatechuic acid alleviates inflammation and EndMT by inhibiting the HDAC1/GATA4 signaling pathway, thereby preserving cardiac function and retarding the progression of myocardial fibrosis. These findings provide a theoretical and experimental foundation for the potential application of protocatechuic acid in treating cardiovascular diseases.

## Simple Summary

This study examines a potential new approach to treating myocardial fibrosis, a major pathology contributing to heart failure. We investigated protocatechuic acid, a natural compound widely distributed in plants with known anti-inflammatory effects, to see if it could ameliorate myocardial fibrosis and elucidate the underlying mechanism. By using animal models, human umbilical vein endothelial cells and human cardiomyocytes, as well as computer simulations, we found that protocatechuic acid ameliorated cardiac function and alleviated myocardial fibrosis through regulating a specific protein pathway in vascular cells. In summary, this natural compound is effective in delaying disease progression by anti-inflammation and protecting heart cells. These promising results indicate that protocatechuic acid is expected to be a new drug candidate for the prevention or treatment of cardiovascular diseases.

## 1. Introduction

Cardiovascular disease imposes a dual burden on global public health and economic systems, attributable to its high prevalence and limited therapeutic options [1,2,3,4]. Myocardial fibrosis is a core pathological mechanism that underlies the progression of a spectrum of cardiovascular diseases, including myocardial infarction, hypertensive heart disease, and diabetic cardiomyopathy, to heart failure [5,6,7,8]. It is characterized by the abnormal activation of cardiac fibroblasts, excessive deposition of extracellular matrix (ECM), and increased myocardial stiffness, resulting in severe impairment of both diastolic and systolic function [9,10]. The communication network between different myocardial cell populations, which is crucial for cardiac damage repair, represents one such overlooked area [11,12,13]. While previous research has predominantly focused on the role of fibroblast activation in myocardial fibrosis [14,15,16,17], the critical contribution of endothelial cells to heart failure progression has been relatively underexplored, especially regarding their paracrine signaling and cellular crosstalk—fundamental processes underlying cardiac repair. Within this complex network, endothelial cells emerge as a central signaling hub, where they serve as key maintainers of cardiovascular structure and function by dynamically regulating vascular tone and the growth of surrounding cells [18]. Beyond the fundamental supply of oxygen and nutrients, overall cardiac function critically depends on the diverse biological signals mediated by endothelial cells via autocrine and paracrine pathways, which are vital for cardiomyocyte development, contractility, post-ischemic survival, and limited regeneration [19,20]; consequently, vascular endothelial dysfunction, which is closely associated with inflammatory responses, is a key driver in the initiation and progression of myocardial fibrosis [21]. Under pathological stimulation, such as chronic inflammation, endothelial cells differentiate into fibroblasts through endothelial–to-mesenchymal transition (EndMT). These cells then secrete large amounts of extracellular matrix into cardiac and perivascular tissues, causing abnormal collagen deposition and ultimately promoting the initiation and progression of myocardial fibrosis [22].

Protocatechuic acid (PCA) is a natural phenolic compound widely distributed in plant-based foods and herbs such as grapes, blueberries, tea, cereals, and *Eucommia ulmoides* [23,24]. Studies have shown that protocatechuic acid exhibits a wide spectrum of pharmacological effects, including anti-inflammatory, antibacterial, and antioxidant properties, as well as neuroprotective effects, such as the potential to mitigate the progression of Alzheimer’s disease [23,25,26,27,28]. In the context of cardiac fibrosis treatment, protocatechuic acid has also shown significant efficacy—for instance, it alleviates isoproterenol-induced cardiac hypertrophy and heart failure in murine models and inhibits angiotensin II (Ang II)-induced activation of cardiac fibroblasts [29,30,31]. Based on these findings, protocatechuic acid holds promise as a potential candidate for myocardial fibrosis therapy, with mechanisms of action likely through mechanisms involving the improvement of vascular endothelial function and modulation of relevant signaling pathways.

The therapeutic potential of protocatechuic acid in mitigating myocardial fibrosis may involve its targeting of specific epigenetic and transcriptional regulators. Studies have shown that histone deacetylase 1 (HDAC1) promotes the release of pro-inflammatory factors (such as TNF-α and IL-6) through epigenetic regulatory mechanisms and activates the nuclear factor-kappa B (NF-κB) signaling pathway, thereby exacerbating inflammatory injury [32,33,34]. On the other hand, GATA binding protein 4 (GATA4), a zinc finger transcription factor [35], plays a central regulatory role in cardiac development and pathological remodeling [36]. Previous research has demonstrated that it promotes cardiomyocyte growth by regulating the expression of various cardiac-specific genes [37,38,39]. Furthermore, GATA4 overexpression significantly suppresses the expression of pro-fibrotic and *TGFB1*-related genes in cardiac fibroblasts [40], suggesting its potential as a broad-spectrum anti-fibrotic factor across diverse cell types. Notably, HDAC1 and GATA4 exhibit a close interaction and jointly regulate downstream gene expression to participate in cardiac pathophysiological processes [41,42].

However, emerging evidence underscores the pivotal role of endothelial cells in the initiation and progression of cardiac fibrosis [43,44]. Specifically, endothelial dysfunction, often marked by a pro-inflammatory and pro-fibrotic phenotypic shift (including EndMT), is a primary event that drives inflammation and the subsequent activation of cardiac fibroblasts through paracrine signaling [45]. It is noteworthy that HDAC1 and GATA4 are also functionally expressed in endothelial cells and may interact to govern endothelial homeostasis. Based on this, we postulate that the HDAC1/GATA4 pathway serves as a critical regulatory node not only in fibroblasts but also, and perhaps more fundamentally, in endothelial cells to control this phenotypic shift.

In this study, we hypothesize that protocatechuic acid exerts synergistic anti-inflammatory and anti-fibrotic effects by modulating HDAC1 activity within endothelial cells, consequently influencing the expression or function of GATA4 to restore endothelial homeostasis. This investigation aims to elucidate the mechanism of protocatechuic acid within this specific environment, providing both a theoretical foundation and experimental support for its development as a therapeutic agent against myocardial fibrosis.

## 2. Materials and Methods

### 2.1. Main Reagents and Drugs

Protocatechuic acid (B21614, purity ≥ 98%) and entinostat (MS-275, S44473, purity ≥ 98%) were purchased from Shanghai yuanye Bio-Technology Co., Ltd. (Shanghai, China). Isoproterenol hydrochloride (R015558) was obtained from RHAWN Reagents (Shanghai, China). Ang II (HY-13948, purity = 99.03%) was sourced from MedChemExpress (MCE) Inc. (Monmouth Junction, NJ, USA). Reactive Oxygen Species Assay Kit (S0033S), Nitric Oxide Assay Kit (S0021S), RIPA Lysis Buffer (P0013B), SDS-PAGE Loading Buffer (5X, P0286), Enhanced BCA Protein Assay Kit (P0010S), and Lipo6000™ Transfection Reagent (C0526) were procured from Beyotime Biotechnology (Shanghai, China). Bovine Serum Albumin (BSA, GC305010), Hematoxylin and Eosin (H&E) Staining Kit (G1005), Masson’s Trichrome Staining Kit (G1006), and Picrosirius Red Staining Kit (G1078) were acquired from Wuhan Servicebio Technology Co., Ltd. (Wuhan, China). Reverse Transcription Kit (BL699A) and ECL Chemiluminescent Substrate Reagent (BL520A) were obtained from Biosharp (Hefei, China). SYBR Green Fast qPCR Mix (RK21203) was purchased from ABclonal (Wuhan, China).

### 2.2. Animals

Sprague-Dawley (SD) rats (250–300 g) were purchased from Shanghai SLAC Laboratory Animal Co., Ltd. (Shanghai, China). The rats were housed in the Animal Experiment Research Center of Zhejiang Chinese Medical University. Prior to experiments, all animals were acclimatized for one week under standard conditions: a controlled environment maintained at 24 ± 1 °C, humidity of 55 ± 5%, a 12 h light/dark cycle, with standard diet and water provided ad libitum. The animal experimental protocol was reviewed and approved by the Institutional Animal Care and Use Committee (IACUC) of Zhejiang Chinese Medical University. The ethics application number is 202502-0164, and the animal experiment approval number is IACUC-20250303-17, and this study adhered to the ARRIVE guidelines.

### 2.3. Model Establishment, Drug Administration, and Grouping

Under aseptic conditions, a rat model of myocardial fibrosis was established via subcutaneous injection of isoproterenol hydrochloride (ISO). ISO was dissolved in normal saline and administered at a dose of 5 mg/kg, while the control (CON) group received an equivalent volume of normal saline. This administration was continued for 7 days. The 7-day ISO administration protocol was selected based on literature demonstrating its efficacy in inducing stable fibrotic pathology [46], The fibrosis induced by this protocol was further validated by our pilot studies, which confirmed that myocardial fibrosis remained stable from day 7 to day 35.

After successful model induction, the rats were divided into five groups (*n* = 5): the CON group, the ISO group, the ISO + protocatechuic acid low-dose group (50 mg/kg, PCA-L), the ISO + protocatechuic acid high-dose group (100 mg/kg, PCA-H), and the ISO + MS-275 group (positive control, an HDAC1 inhibitor). Starting from day 8, the protocatechuic acid treatment groups received daily oral gavage administrations for 28 consecutive days. Specifically, the low (50 mg/kg) and high (100 mg/kg) doses were chosen within the commonly reported effective pharmacological range (typically 25–200 mg/kg) for protocatechuic acid in rodent models, based on previous studies [47,48,49]. The MS-275 treatment group received oral gavage administrations every other day at a dose of 5 mg/kg, also for 28 days, a regimen and dose established in prior research [50,51]. After 28 days of treatment, the rats were euthanized, and cardiac tissue and serum were collected.

### 2.4. Echocardiographic Analysis

After the final administration, rats were placed in an anesthesia induction chamber containing isoflurane for continuous inhalation anesthesia. Echocardiographic examination was performed using the Vevo 1100 system with a 15 MHz transducer (FUJIFILM VisualSonics Inc., Toronto, ON, Canada). Measured parameters included left ventricular ejection fraction (LVEF), left ventricular fractional shortening (LVFS), left ventricular posterior wall thickness at end-diastole (LVPWD), left ventricular end-diastolic volume (LVEDV), and left ventricular end-systolic volume (LVESV). All measurements were recorded over at least three consecutive cardiac cycles.

### 2.5. Biochemical Assays

Serum levels of lactate dehydrogenase (LDH) and creatine kinase isoenzyme-MB (CK-MB) were measured using an automated biochemical analyzer (Hitachi 7020, Tokyo, Japan) with dedicated commercial assay kits (Roche Diagnostics, Basel, Switzerland). The analyzer performed all kinetic measurements and calculations automatically according to the International Federation of Clinical Chemistry (IFCC) standard methods, reporting results in international units per liter (U/L). Based on the Griess reaction, the concentration of nitric oxide (NO) was estimated by measuring its stable metabolites (nitrite and nitrate) using a Nitric Oxide Assay Kit (Beyotime Biotechnology, Cat# S0021, Shanghai, China) following the manufacturer’s protocol. The concentration (μM) was calculated based on a standard curve of sodium nitrite (0–100 μM).

### 2.6. Histological Analysis

Heart tissues were collected and fixed in 4% paraformaldehyde, followed by dehydration, embedding in paraffin, and sectioning into 5 μm thick slices. Paraffin-embedded heart sections were subjected to Hematoxylin and Eosin (H&E) staining to observe morphological characteristics of the damaged tissue, as well as Masson’s trichrome and picro-sirius red staining to assess the extent of fibrosis. Slides were scanned and imaged using an automated digital slide scanner (C13210-01, Hamamatsu, Japan) and a polarized light microscope (Eclipse Ci POL, Tokyo, Japan). Type I and Type III collagen fibers were distinguished based on their birefringence colors under polarized light.

Quantitative analysis was conducted using ImageJ software (version 1.54g). The inflammatory area fraction was determined from H&E images using the formula: (area of inflammatory foci/total myocardial area) × 100%. Fibrosis was quantified by calculating the total collagen volume fraction (CVF) with the “Color Threshold” tool: (collagen-positive area/total myocardial area) × 100%. Additionally, the type I/III collagen ratio was determined by applying the same tool to polarized picro-sirius red images to distinguish between the red/yellow birefringence of type I collagen and the green birefringence of type III collagen.

### 2.7. Immunohistochemistry

Heart sections (5 μm) were deparaffinized, rehydrated, and subjected to antigen retrieval in citrate buffer (pH 6.0) via microwave heating. Endogenous peroxidase was blocked with 3% H_2_O_2_, followed by serum blocking and overnight incubation with primary antibodies at 4 °C. After HRP-conjugated secondary antibody incubation, DAB was used for visualization, with hematoxylin counterstaining. Imaging and quantification were performed using ImageJ. Antibody details are listed in Table 1.

### 2.8. Cell Culture and Treatment

Human umbilical vein endothelial cells (HUVECs) and AC16 human cardiomyocytes were purchased from Procell (Wuhan, China). HUVECs were cultured in ECM medium, and AC16 cells were maintained in Dulbecco’s Modified Eagle Medium (DMEM). Both media were supplemented with 10% fetal bovine serum (FBS) and 1% penicillin/streptomycin. All cells were incubated at 37 °C in a humidified atmosphere containing 5% CO_2_. The culture medium was replaced every 1 to 2 days. Upon reaching approximately 80% confluence, the cells were detached using 0.25% trypsin and passaged for subsequent experiments.

HUVECs in the optimal growth state were randomly divided into 6 groups: (1) CON group: cells cultured under normal conditions; (2) Ang II group: cells treated with 1 μM Ang II for 24 h, followed by replacement with normal culture medium; (3) PCA-L group: cells treated with 1 μM Ang II for 24 h, followed by treatment with 25 μM protocatechuic acid for 24 h; (4) PCA-M group: cells treated with 1 μM Ang II for 24 h, followed by treatment with 50 μM protocatechuic acid for 24 h; (5) PCA-H group: cells treated with 1 μM Ang II for 24 h, followed by treatment with 100 μM protocatechuic acid for 24 h.

### 2.9. Immunofluorescence Staining

Cells were fixed with 4% paraformaldehyde (15 min, RT), washed with PBS, permeabilized with 0.3% Triton X-100/PBS (20 min), and blocked with 5% BSA (1 h, RT). After incubation with primary antibodies overnight at 4 °C, cells were washed and incubated with a CoralLite Plus 594-conjugated goat anti-rabbit recombinant secondary antibody (1:200, 1 h, light-protected). Nuclei were stained with DAPI-containing anti-fade mounting medium (10 min) before imaging. Quantitative analysis was performed using ImageJ software.

### 2.10. Real-Time Quantitative PCR (RT-qPCR)

Total RNA extraction and cDNA synthesis followed the respective kit instructions. RT-qPCR was performed using SYBR Green Fast qPCR Mix under the protocol: 95 °C for 3 min; 40 cycles of 95 °C for 5 s and 60 °C for 30 s. GAPDH was used as the internal reference, and relative gene expression was calculated by the 2^−ΔΔCt^ method (control group as calibrator). All primer sequences are provided in Table 2.

### 2.11. Protein Extraction and Western Blotting

Proteins were extracted using RIPA lysis buffer, followed by centrifugation to collect the supernatant. Protein concentration was measured with a BCA assay kit. Equal amounts of proteins were separated by 10% SDS-PAGE and transferred to polyvinylidene fluoride (PVDF, ISEQ00005, Millipore, Billerica, MA, USA) membranes. After blocking with 5% BSA for 2 h at room temperature, the membranes were incubated with primary antibodies overnight at 4 °C, followed by incubation with corresponding secondary antibodies for 1 h at room temperature. Protein bands were visualized using an ECL substrate on an Azure Biosystems imaging system (Azure 300, Dublin, CA, USA).

### 2.12. Co-Immunoprecipitation (Co-IP)

For Co-IP, a specific primary antibody (with an isotype IgG as a control) was pre-adsorbed onto Protein A/G magnetic beads. The bead-antibody complexes were then incubated with HUVEC lysates at 4 °C overnight to capture the target protein complexes. Following extensive washing, the immunoprecipitated complexes were eluted by boiling in SDS loading buffer and subsequently analyzed by Western blotting to detect the co-precipitated target proteins.

### 2.13. siRNA Transfection

The siRNAs targeting HDAC1 and the negative control siRNA were purchased from Sangon Biotech Co., Ltd. (Shanghai, China). Transfection experiments were performed using Lipo6000™ transfection reagent (Beyotime Biotechnology). The day prior to transfection, cells were seeded in 6-well plates at a density of 2 × 10^5^ cells per well to reach approximately 40–50% confluence. For each well, a transfection complex was prepared by mixing 100 pmol of siRNA with 5 μL of Lipo6000™ reagent in 250 μL of serum-free Opti-MEM medium, followed by incubation at room temperature. The complex was then added to the culture wells. After 6 h, the medium was replaced with complete growth medium. Silencing efficiency was assessed by Western blot 48 h post-transfection.

### 2.14. Molecular Docking

The three-dimensional structure of protocatechuic acid was obtained from the PubChem database, while the protein structure file of HDAC1 was sourced from the Protein Data Bank (PDB). Following the methodology described by Liu et al. [46], we prepared the 3D structure of the protocatechuic acid and the crystal structure of the HDAC1 using AutoDock Tools (version 1.5.6). Molecular docking was subsequently performed using AutoDock Vina (version 1.2.3). Finally, visualization and analysis were conducted using PyMOL 3.0 and Discovery Studio 2019.

### 2.15. Molecular Dynamics Simulation

Upon completion of molecular docking, molecular dynamics simulation was performed using Gromacs 2022 software with the AMBER ff14SB force field and the TIP3P water model to validate the stability of the complex conformation exhibiting the lowest binding energy. The complex was solvated in a dodecahedron box, maintaining a minimum distance of 1.2 nm between any protein atom and the box boundary. Energy minimization was conducted using the steepest descent algorithm until the system energy converged to a tolerance of 1000 kJ·mol^−1^·nm^−1^. Finally, various analytical scripts integrated within GROMACS were employed to process and analyze the simulation trajectories.

### 2.16. Calculation of Binding Energy Using MM-PBSA

The binding free energy was calculated for the last 20 ns segment of each trajectory using the gmx_MMPBSA tool. This method, based on the Molecular Mechanics/Poisson-Boltzmann Surface Area (MM/PBSA) approach, provides a precise assessment of the binding free energy between the ligand and the receptor.

### 2.17. Statistical Analysis

Statistical analysis was performed using SPSS 25.0 software. The normality of data distribution for each group was assessed using the Shapiro–Wilk test. Experimental data are expressed as mean ± standard deviation. Comparisons between two groups were conducted using either the unpaired two-tailed Student’s *t*-test (for normally distributed data) or the Mann–Whitney U test (for non-normally distributed data). Comparisons among multiple groups were performed using one-way ANOVA (for normally distributed data with homogeneity of variance) followed by Tukey’s post hoc test, or the Kruskal–Wallis test (for non-normally distributed data) followed by Dunn’s post hoc test. *p* values less than 0.05 were considered statistically significant.

Declaration of GenAI use: The authors used DeepSeek V3.2 (Hangzhou DeepSeek Artificial Intelligence Co., Ltd., Hangzhou, China) solely for language polishing and grammar checking during the preparation of this work.

## 3. Results

### 3.1. Protocatechuic Acid Treatment Attenuates Isoproterenol-Induced Cardiac Dysfunction in Rats

**Figure 1:**
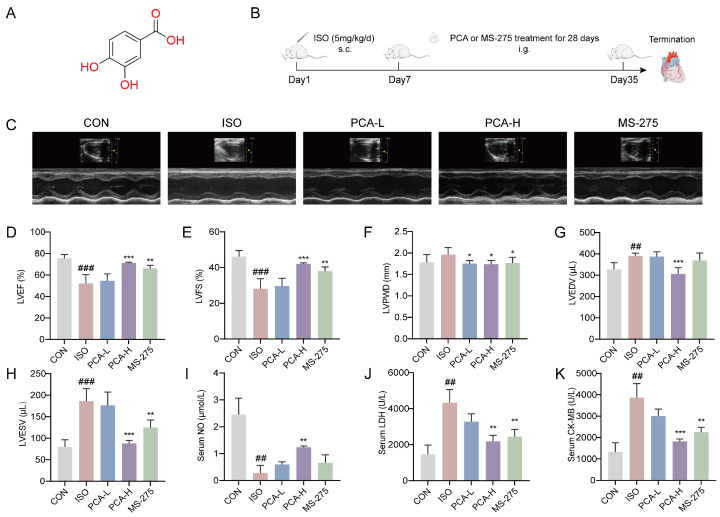
Protocatechuic acid alleviates cardiac function injury in rats. (**A**) Chemical structure of protocatechuic acid. (**B**) Schematic diagram of the animal experimental procedure. (**C**) Representative M-mode echocardiographic images from different groups. (**D**–**H**) Echocardiographic parameters of SD rats (*n* = 5): (**D**) left ventricular ejection fraction (LVEF), (**E**) left ventricular fractional shortening (LVFS), (**F**) left ventricular end-diastolic volume (LVEDV), (**G**) left ventricular end-systolic volume (LVESV) and (**H**) left ventricular posterior wall thickness at end-diastole (LVPWD). (**I**–**K**) Expression levels of (**I**) nitric oxide (NO), (**J**) lactate dehydrogenase (LDH), and (**K**) creatine kinase-MB (CK-MB) in the serum of SD rats (*n* = 3). ^##^ *p* < 0.01, ^###^ *p* < 0.001 vs. CON group; * *p* < 0.05, ** *p* < 0.01, *** *p* < 0.001 vs. ISO group.

To evaluate the cardioprotective effects of protocatechuic acid in a rat model of ISO-induced myocardial fibrosis, we assessed cardiac function using echocardiography and measured relevant serum biomarkers. Figure 1A,B depicts the structural formula of protocatechuic acid, the experimental model setup, and the drug administration regimen. To further elucidate the pharmacological profile of protocatechuic acid, the HDAC1-specific inhibitor entinostat (MS-275, 5 mg/kg) was included as a positive control in addition to the standard experimental groups. Echocardiographic results revealed that compared to the CON group, rats in the ISO group exhibited significant cardiac impairment, characterized by a marked reduction in left ventricular ejection fraction (LVEF) and left ventricular fractional shortening (LVFS), alongside significant increases in left ventricular end-diastolic volume (LVEDV) and left ventricular end-systolic volume (LVESV). Left ventricular posterior wall thickness at end-diastole (LVPWD) showed an increasing trend in the ISO group, although the difference compared to the CON group was not statistically significant. Following a four-week intervention, treatment with 100 mg/kg protocatechuic acid significantly improved the impaired cardiac function parameters, demonstrating a notable increase in LVEF and LVFS, along with a significant decrease in LVEDV and LVESV. In the MS-275 (5 mg/kg) group, LVEDV showed a decreasing trend compared to the ISO group, although this did not reach statistical significance. Administration of 50 mg/kg protocatechuic acid also showed a tendency towards improvement in these parameters, but the effects did not reach statistical significance compared to the ISO group (Figure 1C–H).

In terms of serum biomarker detection, compared to the CON group, rats in the ISO group exhibited a significant decrease in serum nitric oxide (NO) levels, along with a significant increase in lactate dehydrogenase (LDH) and creatine kinase-MB (CK-MB) levels. After four weeks of treatment, the 100 mg/kg protocatechuic acid group showed elevated serum NO levels and reduced levels of myocardial injury biomarkers LDH and CK-MB. In the MS-275 group, serum NO levels showed an increasing trend compared to the ISO group, though the change was not statistically significant. The 50 mg/kg protocatechuic acid group did not exhibit statistically significant improvements in these serum biomarkers compared to the ISO group (Figure 1I–K).

These results suggest that protocatechuic acid, at the dose of 100 mg/kg, exerts cardioprotective effects by mitigating cardiomyocyte injury and improving cardiac function in this model.

### 3.2. Protocatechuic Acid Treatment Alleviates Isoproterenol-Induced Cardiac Pathological Damage and Suppresses Collagen Fiber Deposition in Rats

**Figure 2:**
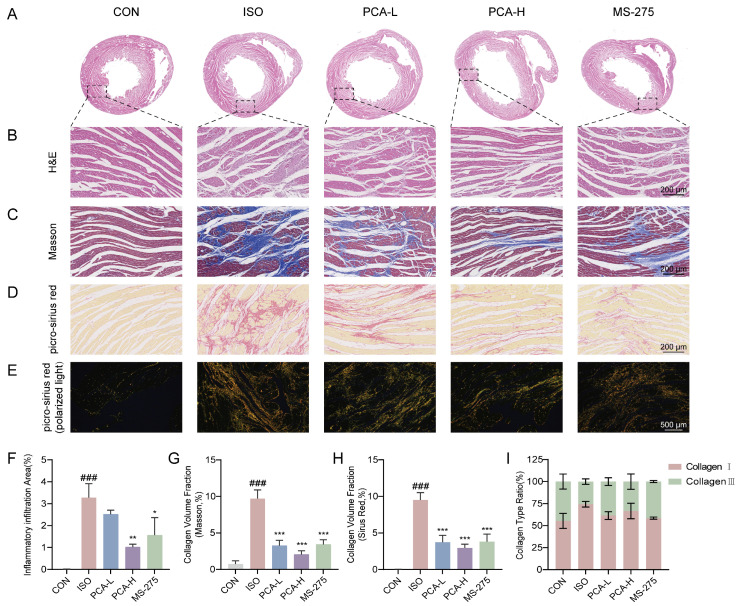
Protocatechuic acid alleviates pathological cardiac injury and suppresses collagen fiber deposition in rats. (**A**,**B**) Representative H&E staining images of cardiac tissues. Scale bar: 200 μm. (**C**) Representative Masson’s trichrome staining images of cardiac tissues. Scale bar: 200 μm. (**D**) Representative picro-sirius red staining images of cardiac tissues. Scale bar: 200 μm. (**E**) Polarized light images of picro-sirius red-stained cardiac tissues: Collagen type I fibers appear red/yellow, and Collagen type III fibers appear green. Scale bar: 500 μm. (**F**) Quantitative analysis of inflammatory cell infiltration areas from H&E-stained myocardial sections (*n* = 3). (**G**,**H**) Statistical analysis of the collagen volume fraction (CVF) from Masson’s trichrome and picro-sirius red staining in myocardial tissues (*n* = 3). (**I**) Statistical analysis of the area percentage of Collagen type I and Collagen type III from polarized light images of picro-sirius red staining (*n* = 3). ^###^ *p* < 0.001 vs. CON group; * *p* < 0.05, ** *p* < 0.01, *** *p* < 0.001 vs. ISO group.

To further validate the cardioprotective effects at the tissue level, histopathological analysis was performed. H&E staining results revealed clear and intact myocardial tissue structure, well-arranged muscle fibers, and no significant pathological changes in the CON group. In contrast, the ISO group exhibited significant myocardial necrosis, disruption of muscle fibers, and extensive inflammatory cell infiltration. After intervention with 100 mg/kg protocatechuic acid or 5 mg/kg MS-275, the ISO-induced myocardial histopathological damage was markedly ameliorated, with the myocardial tissue structure largely restored to normal or showing only mild pathological alterations. Notably, while the 50 mg/kg protocatechuic acid treatment (PCA-L group) showed a trend toward improvement compared to the ISO group, the difference was not statistically significant (Figure 2A,B). In addition, the anti-fibrotic effects of protocatechuic acid were evaluated by assessing myocardial collagen deposition with Masson’s trichrome and picro-sirius red staining. As shown in Figure 2C,D, compared with the CON group, the ISO group showed a significant increase in the degree of myocardial interstitial fibrosis. However, treatment with different doses of protocatechuic acid (50 mg/kg or 100 mg/kg) or MS-275 (5 mg/kg) significantly reduced myocardial collagen deposition. Furthermore, picro-sirius red staining under polarized light microscopy was used to distinguish type I collagen (red/yellow birefringence) from type III collagen (green birefringence). Representative images (Figure 2E) visually demonstrated that the excessive accumulation of both collagen types induced by ISO was effectively suppressed by intervention with 100 mg/kg protocatechuic acid or MS-275. These findings collectively confirm that protocatechuic acid at a dose of 100 mg/kg can effectively ameliorate the pathological progression of myocardial fibrosis by regulating collagen expression and deposition.

### 3.3. Protocatechuic Acid Inhibits Inflammation and Endothelial–Mesenchymal Transition, as Well as Regulates the Expression of Histone Deacetylase 1 and GATA Binding Protein 4

EndMT serves as a significant source of cardiac fibroblasts. Under ischemic and hypoxic conditions, endothelial cells undergo EndMT, differentiating into fibroblasts that secrete excessive extracellular matrix into myocardial and perivascular tissues, thereby promoting collagen deposition and cardiac fibrosis. To investigate the underlying molecular mechanisms, we performed immunohistochemical analysis of α-SMA, CD31, HDAC1, and GATA4 on cardiac tissue sections.

**Figure 3:**
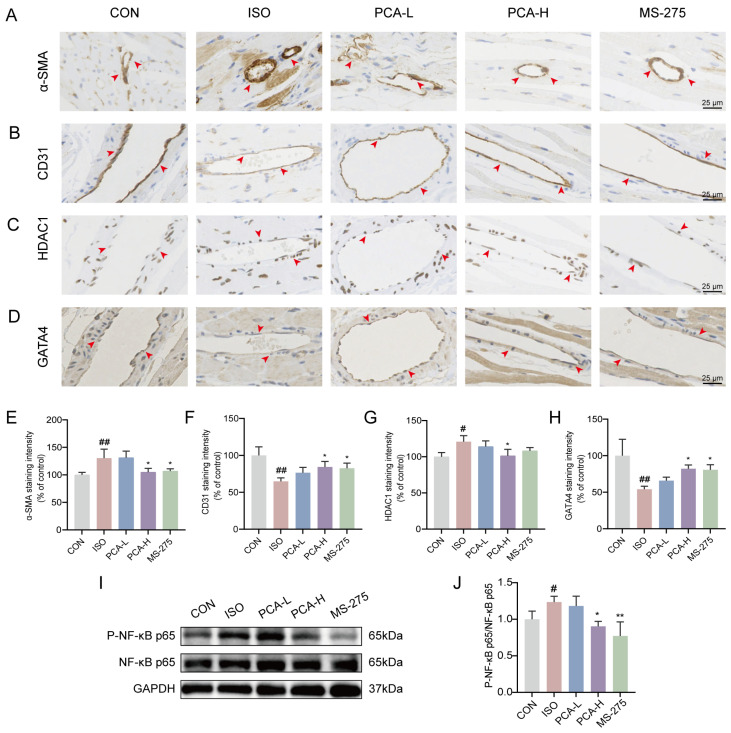
Protocatechuic acid inhibits inflammation and endothelial–mesenchymal transition, as well as regulates the expression of histone deacetylase 1 and GATA binding protein 4. (**A**–**D**) Representative immunohistochemical staining images of α-SMA, CD31, HDAC1, and GATA4 proteins in cardiac tissues. Brown color indicates positive signals (as indicated by arrows). Scale bars: 25 μm. (**E**–**H**) Statistical analysis of immunohistochemical staining intensity for α-SMA, CD31, HDAC1, and GATA4 proteins (*n* = 3). (**I**) Representative Western blot images of NF-κB p65, P-NF-κB p65 and GAPDH. (**J**) Quantitative analysis of P-NF-κB p65 protein expression by western blot (*n* = 3). ^#^ *p* < 0.05, ^##^ *p* < 0.01 vs. CON group; * *p* < 0.05, ** *p* < 0.01 vs. ISO group.

The results showed a significant increase in α-SMA staining in the vascular wall and surrounding tissues in the ISO group compared with the CON group (Figure 3A,E), indicating fibrosis activation. Following intervention with protocatechuic acid (100 mg/kg) and MS-275 (5 mg/kg), α-SMA expression was reduced, suggesting amelioration of fibrosis (Figure 3A,E).

As a marker of endothelial integrity, CD31 exhibited a continuous and intact distribution along the vascular endothelium in the CON group, whereas its expression was substantially reduced in the ISO group (Figure 3B,F). Treatment with protocatechuic acid (100 mg/kg) and MS-275 partially restored CD31 expression. However, the effect of protocatechuic acid at 50 mg/kg was not statistically significant compared with the ISO group (Figure 3B,F).

Furthermore, compared with the CON group, the ISO group showed upregulated expression of HDAC1 but downregulated expression of GATA4 in perivascular cells and tissues. Protocatechuic acid (100 mg/kg) effectively reversed these alterations (Figure 3C,D,G,H). Notably, although protocatechuic acid at 50 mg/kg modulated the expression trends of these markers, the changes were not statistically significant relative to the ISO group. For HDAC1, MS-275 treatment showed a regulatory trend similar to that of protocatechuic acid (100 mg/kg), but it did not reach statistical significance.

To further validate the anti-inflammatory effects of protocatechuic acid, Western blot analysis was performed. The results revealed a significant increase in the expression of phosphorylated NF-κB p65 (P-NF-κB p65) in the ISO group compared with the CON group, indicating the activation of inflammatory responses. Both protocatechuic acid (100 mg/kg) and MS-275 effectively reversed this alteration in protein expression (Figure 3I,J), (For full-length, uncropped western blots, see File S1), suggesting that protocatechuic acid exerts an anti-inflammatory effect through modulation of the NF-κB signaling pathway.

### 3.4. Protocatechuic Acid Inhibits Angiotensin II-Induced Endothelial Inflammation Through the Histone Deacetylase 1/GATA Binding Protein 4 (HDAC1/GATA4) Pathway

**Figure 4:**
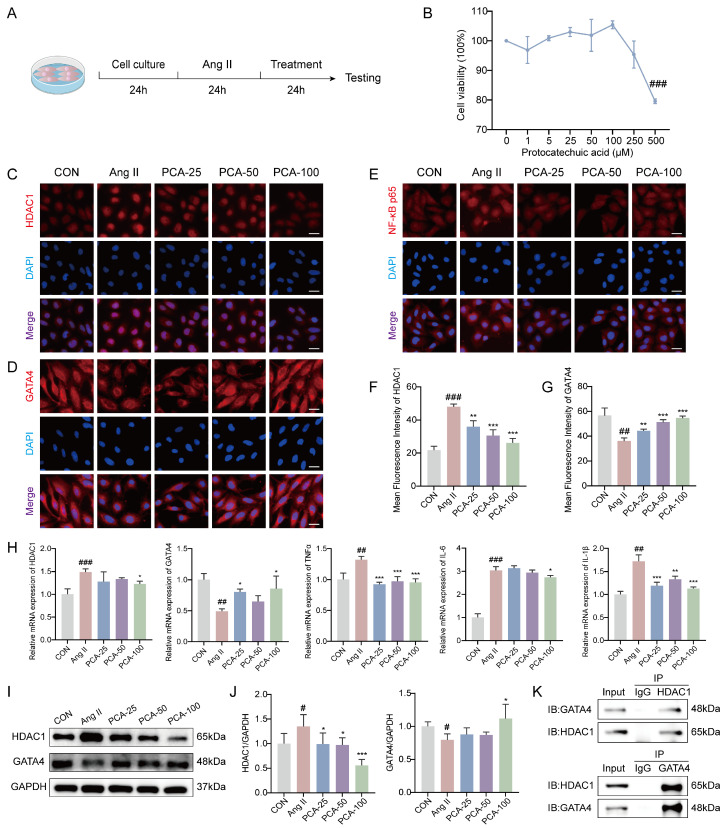
Protocatechuic acid inhibits angiotensin II-induced endothelial inflammation through the histone deacetylase 1/GATA binding protein 4 (HDAC1/GATA4) pathway. (**A**) Diagram of the cell experiment design. (**B**) Cell viability of HUVECs treated with various concentrations of protocatechuic acid (*n* = 3). (**C**–**E**) Immunofluorescence staining images of HDAC1, GATA4, and NF-κB p65 proteins in HUVECs. Red fluorescence represents the target protein signal, and blue fluorescence (DAPI) represents the nucleus. Scale bar: 25 μm. (**F**,**G**) Quantitative analysis of the mean fluorescence intensity of HDAC1 and GATA4 in HUVECs (*n* = 3). (**H**) mRNA levels of *HDAC1*, *GATA4*, *TNF-α*, *IL-6*, and *IL-1β* in HUVECs detected by RT-qPCR (*n* = 3). (**I**) Representative Western blot images of HDAC1, GATA4 and GAPDH. (**J**) Quantitative analysis of HDAC1 and GATA4 protein expression by western blot (*n* = 3). (**K**) Endogenous Co-IP analysis of HDAC1 and GATA4. Co-IP (IP: α-HDAC1), IB: as indicated. Reverse Co-IP (IP: α-GATA4), IB: as indicated. IgG served as a negative control. ^#^ *p* < 0.05, ^##^ *p* < 0.01, ^###^ *p* < 0.001 vs. CON group; * *p* < 0.05, ** *p* < 0.01, *** *p* < 0.001 vs. Ang II group.

To evaluate the effect of protocatechuic acid on the viability of HUVECs, we performed CCK-8 assays after treating the cells with different concentrations of PCA (0–500 μM) for 24 h. The results (Figure 4B) demonstrated that PCA exhibited significant cytotoxicity at 500 μM, while cell viability remained high at concentrations of 25, 50, and 100 μM. Accordingly, the concentrations of 25, 50, and 100 μM were selected for subsequent experiments. Following the determination of the non-cytotoxic working concentrations, we further investigated whether protocatechuic acid influences EndMT and fibrosis progression by modulating the inflammatory response mediated by the HDAC1/GATA4 signaling pathway. For this analysis, based on prior research, we selected HDAC1, GATA4, NF-κB p65, and the pro-inflammatory factors TNF-α, IL-6, and IL-1β as key effector molecules. Immunofluorescence results showed that Ang II stimulation significantly upregulated HDAC1 protein expression, downregulated GATA4 protein expression, and promoted p65 nuclear translocation, whereas protocatechuic acid treatment markedly inhibited these effects (Figure 4C–G). This suggests that protocatechuic acid may exert its anti-inflammatory effects by suppressing NF-κB pathway activation via the HDAC1/GATA4 signaling pathway. RT-qPCR results indicated that Ang II induction significantly upregulated the mRNA levels of pro-inflammatory factors (*TNF-α*, *IL-6*, *IL-1β*) and *HDAC1* in HUVECs, accompanied by a downregulation of *GATA4* transcription. Protocatechuic acid treatment reversed these gene expression changes (Figure 4H). Western blot results further confirmed that HDAC1 protein expression was significantly increased and GATA4 protein expression decreased in the Ang II-treated group, while protocatechuic acid treatment counteracted these alterations (Figure 4I,J), consistent with the transcriptional trends. Additionally, co-immunoprecipitation results suggested an interaction between HDAC1 and GATA4 (Figure 4K). Collectively, these results demonstrate that protocatechuic acid attenuates NF-κB-driven inflammatory responses and improves the vascular endothelial microenvironment through regulation of the HDAC1/GATA4 signaling pathway.

### 3.5. Histone Deacetylase 1 Knockdown and Protocatechuic Acid Intervention Inhibit Endothelial Inflammation and Fibrotic Process Through Histone Deacetylase 1/GATA Binding Protein 4 (HDAC1/GATA4) Pathway

**Figure 5:**
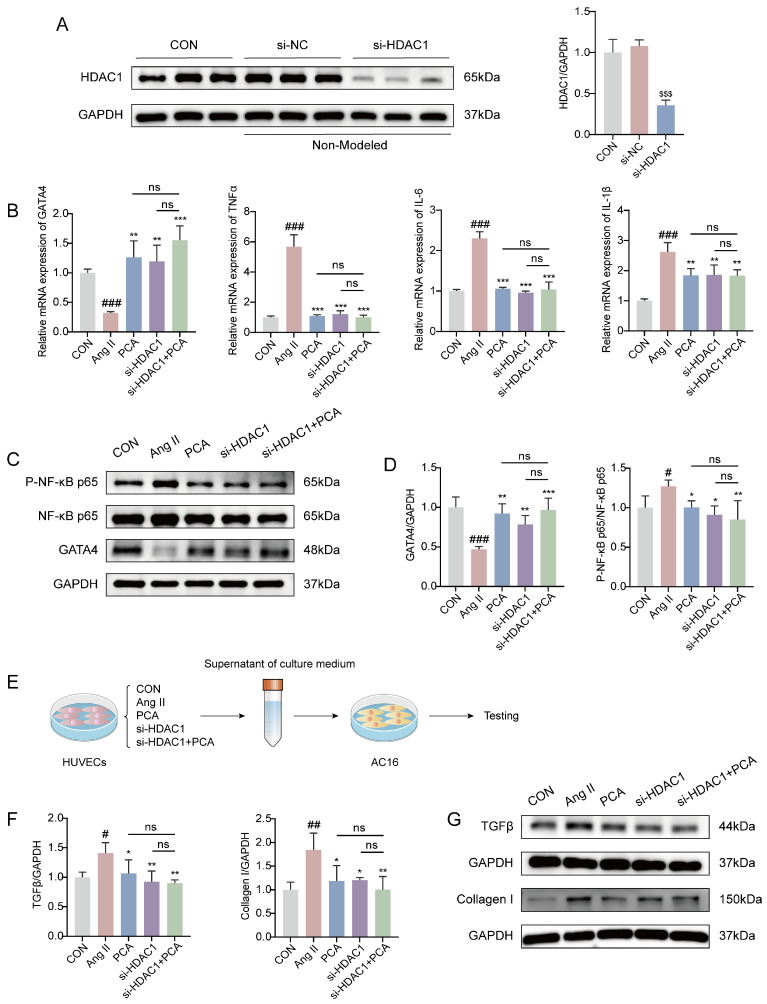
Histone deacetylase 1 knockdown and protocatechuic acid intervention inhibit endothelial inflammation and fibrotic process through histone deacetylase 1/GATA binding protein 4 (HDAC1/GATA4) pathway. (**A**) Western blot analysis of HDAC1 protein levels in HUVECs from the Con group, si-NC group, and si-HDAC1-transfected group (*n* = 3). (**B**) mRNA levels of *GATA4*, *TNF-α*, *IL-6* and *IL-1β* in HUVECs measured by RT-qPCR (*n* = 3). (**C**) Representative Western blot images of NF-κB p65, P-NF-κB p65, GATA4 and GAPDH. (**D**) Quantitative analysis of P-NF-κB p65 and GATA4 protein expression by Western blot (*n* = 3). (**E**) The co-culture model of HUVECs and AC16 cells. (**F**) Quantitative analysis of TGFβ and Collagen I protein expression by western blot (*n* = 3). (**G**) Representative Western blot images of TGFβ, Collagen I and GAPDH. In (**A**), ^$$$^ *p* < 0.001 vs. si-NC group; in (**B**,**D**,**F**), ^#^ *p* < 0.05, ^##^ *p* < 0.01, ^###^ *p* < 0.001 vs. CON group; * *p* < 0.05, ** *p* < 0.01, *** *p* < 0.001 vs. Ang II group, with ns indicating no statistically significant difference.

To further investigate the impact of HDAC1 on EndMT and the fibrotic process in HUVECs, and based on previous experimental findings indicating that the PCA-100 group exhibited superior intervention effects on relevant pathways, this study selected this dosage as a reference for subsequent mechanistic exploration. HUVECs were transfected with si-NC (negative control) or si-HDAC1. Western blot analysis confirmed that transfection with si-HDAC1 was successful, while no significant difference was observed between the CON and si-NC groups. In contrast, HDAC1 expression was significantly reduced in the si-HDAC1 transfection group compared to the si-NC group (Figure 5A), indicating successful knockdown of HDAC1 by si-HDAC1, with an interference efficiency of approximately 60–70%, meeting the requirements for subsequent experiments. Further RT-qPCR analysis revealed that in Ang II-treated HUVECs, both protocatechuic acid treatment and HDAC1 knockdown downregulated the mRNA expression of pro-inflammatory factors *TNF-α*, *IL-6*, and *IL-1β*, while upregulating the mRNA level of the transcription factor *GATA4* (Figure 5B). To verify whether these changes in gene expression were consistently reflected at the protein level, Western blot analysis was performed. The results demonstrated that GATA4 protein expression was significantly reduced in the Ang II-treated group, whereas the expression of phosphorylated NF-κB p65 (P-NF-κB p65) was markedly increased. Both protocatechuic acid treatment and HDAC1 knockdown reversed these alterations in protein expression. Notably, although the combined intervention of protocatechuic acid and HDAC1 knockdown exhibited a stronger trend in modulating the expression of GATA4 and P-NF-κB p65, the difference did not reach statistical significance compared to either single intervention (Figure 5C,D). Subsequently, AC16 cells were co-cultured with conditioned medium from the HUVECs cultured under the above conditions (Figure 5E), and western blot analysis was performed on the AC16 cells. The results demonstrated that conditioned medium from HUVECs subjected to either protocatechuic acid treatment or HDAC1 knockdown reversed the changes in protein expression of the fibrotic effector molecules TGF-β and Collagen I in AC16 cells, with no significant difference observed compared to the combined intervention of protocatechuic acid and HDAC1 knockdown (Figure 5F,G). These findings suggest that the HDAC1/GATA4 signaling pathway may serve as a key molecular mechanism through which protocatechuic acid inhibits vascular endothelial inflammation and the subsequent fibrotic process, thereby exerting anti-myocardial fibrotic effects.

### 3.6. Integrated Computational Simulations Demonstrate the High Affinity and Stability of Protocatechuic Acid Binding to Histone Deacetylase 1

**Figure 6:**
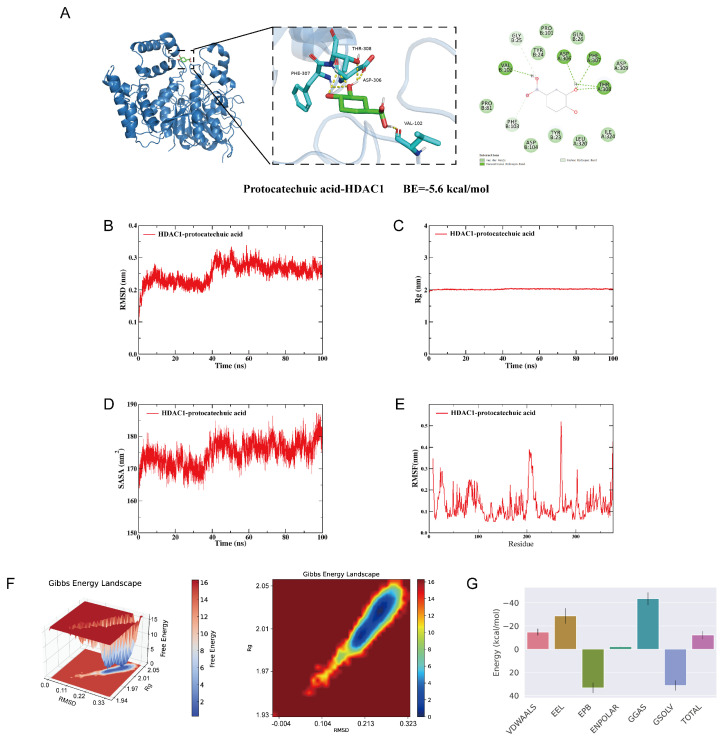
Integrated computational simulations demonstrate the high affinity and stability of protocatechuic acid binding to histone deacetylase 1 (HDAC1). (**A**) 2D and 3D binding models of HDAC1 with protocatechuic acid. (**B**) Root mean square deviation (RMSD) of the complex. (**C**) Radius of gyration (Rg) of the complex. (**D**) Solvent-accessible surface area (SASA) of the complex. (**E**) Root mean square fluctuation (RMSF) of the complex. (**F**) 3D and 2D free energy landscapes for the complex. (**G**) Energy component decomposition for the complex.

Building upon our findings regarding the cardioprotective effects of protocatechuic acid, molecular docking and molecular dynamics simulations were employed to investigate its binding to HDAC1. A lower molecular docking score indicates higher affinity between the ligand and the receptor. Our result showed that the HDAC1-protocatechuic acid complex had a binding energy of approximately −5.6 kcal/mol, which suggests a stable and favorable interaction. Specifically, in its binding to HDAC1 (Figure 6A), protocatechuic acid formed conventional hydrogen bonds with VAL102, ASP306, PHE307, and THR308. Additionally, it established carbon-hydrogen bonds with PHE103 and GLY25 while engaging in van der Waals interactions with LEU320, ASP309, GLN26, TYR24, PRO101, PRO81, ASP104, TYR23, and ILE324. In summary, protocatechuic acid demonstrates strong binding affinity for HDAC1, which is likely to play a key role in regulating fibrosis.

Root mean square deviation (RMSD) is a key metric for evaluating the conformational stability of protein-ligand complexes, reflecting the deviation of atomic positions from the initial structure. Lower RMSD values indicate smaller conformational fluctuations and greater stability of the complex system. As shown in Figure 6B, the HDAC1-protocatechuic acid complex reached equilibrium after 40 ns, with RMSD value stabilizing at approximately 0.25 nm. Overall, protocatechuic acid demonstrated high conformational stability upon binding to the target protein HDAC1. Further analysis revealed that the radius of gyration (Rg) and solvent-accessible surface area (SASA) of the HDAC1-protocatechuic acid complex system remained stable throughout the simulation (Figure 6C,D), indicating that no significant structural contraction or expansion occurred during the molecular dynamics simulation, and the overall conformation remained relatively compact. Additionally, root mean square fluctuation (RMSF) analysis reflects the flexibility of protein residues. As illustrated in Figure 6E, the RMSF value for the HDAC1-protocatechuic acid complex was low, indicating low flexibility of the amino acid residues and high structural rigidity, further confirming the high stability of the complex system.

To investigate the conformational changes within the system, this study employed the free energy landscape (FEL) analysis method. This approach calculates the Gibbs free energy based on RMSD and Rg, through the visualization of free energy variations, provides an in-depth analysis of the molecular energy landscape characteristics and the interaction mechanisms between proteins and ligands. The energy minima in the plots correspond to stable conformational states, while the energy maxima represent energy barriers during conformational transitions. This analysis can effectively predict ligand binding affinity and reveal molecular recognition mechanism. As shown in Figure 6F, the lowest free energy (LFE) minimum for the HDAC1-protocatechuic acid complex is located at RMSD = 0.26 nm and Rg = 2.02 nm. Furthermore, based on the binding conformation, we used the MM/PBSA method to calculate the binding free energy. A more negative binding free energy generally indicates greater complex stability. The result shows that the binding free energy for the HDAC1-protocatechuic acid complex is −12.21 kJ/mol (Figure 6G). These results indicate that protocatechuic acid binds to HDAC1 spontaneously, forming a stable complex.

## 4. Discussion

Cardiovascular disease (CVD) is a major contributor to global mortality and excessive healthcare costs. Despite multifaceted advancements in preventive strategies and treatment approaches, factors such as unhealthy health behaviors and environmental pollution continue to undermine the effectiveness of these interventions. Consequently, the overall mortality rate associated with CVD has maintained an upward trend over the past three decades. Cardiac fibrosis represents a common pathological basis for all cardiovascular diseases, in which endothelial cell populations play a critical role [52]. Persistent exposure to harmful stimuli may induce endothelial dysfunction, which manifests as chronic inflammation, dysregulated secretion of vasoactive factors, or cell death, thereby triggering EndMT and contributing to the onset and progression of various CVDs [53]. A growing body of evidence indicates that suppressing myocardial fibrosis is an effective approach to prevent the progression of heart failure [54]. While current mainstay therapies, such as ACE inhibitors, effectively target the renin–angiotensin–aldosterone system (RAAS), they are associated with adverse effects, including hypotension, proteinuria, and hyperkalemia [55,56]. These limitations underscore the imperative for developing novel therapeutic strategies to address the current clinical dilemma.

In recent years, natural products have emerged as a research hotspot in cardiovascular diseases due to their multi-target effects, low toxicity, and broad range of biological activities, such as antioxidant, anti-inflammatory, antithrombotic, and blood circulation-promoting properties, demonstrating significant potential for application [49,57,58]. In this study, we evaluated the role of protocatechuic acid in cardiac fibrosis. Previous research has shown that protocatechuic acid, a natural phenolic acid compound, exhibits a wide range of biological activities in various disease models [59,60]. Moreover, in terms of cardioprotection, protocatechuic acid has been found to suppress isoproterenol-induced cardiac hypertrophy and oxidative stress [30]. Our findings indicate that protocatechuic acid treatment significantly improves cardiac function in rats with myocardial fibrosis, specifically manifested as a marked increase in LVEF and LVFS, along with a significant reduction in LVPWD, LVEDV, and LVESV. Furthermore, protocatechuic acid effectively inhibited the release of myocardial injury markers LDH and CK-MB in the serum and promoted an increase in serum NO levels. Histopathological analysis further revealed that protocatechuic acid treatment markedly alleviated inflammatory injury in myocardial tissue, as evidenced by reduced inflammatory cell infiltration and diminished structural damage. Additionally, it suppressed the abnormal deposition of collagen I and III and the expression of the mesenchymal marker α-SMA, and promoted the upregulation of the endothelial junction protein CD31, suggesting its positive role in inhibiting the fibrotic process and improving microvascular integrity. In our study, the anti-fibrotic effect of protocatechuic acid reached statistical significance only at the high dose (100 mg/kg), but not at the low dose (50 mg/kg). This indicates a clear dose-effect threshold for its efficacy and underscores the need to define its therapeutic window. Although prior studies have reported good short-term safety for protocatechuic acid, whether long-term high-dose administration could induce hepatorenal abnormalities or other toxicities remains unclear, warranting further evaluation through systematic long-term toxicity experiments.

HDAC1 is primarily localized in the nucleus and plays a central role in maintaining cellular homeostasis. Generally, HDAC1 not only directly regulates transcription through the deacetylation of histones and transcription factors but also modifies various non-histone substrates, directly modulating their enzymatic activity. This enables HDAC1 to precisely mediate both acute responses and chronic adaptations of cells to environmental stimuli [61]. Recent studies have shown that inhibiting HDAC1 activity has a protective effect in various models of myocardial injury [62,63,64]. For example, inhibition of HDAC1 can restore cardiac dysfunction in septic mice [65]. Following myocardial infarction, HDAC1 inhibition has been shown to improve cardiac remodeling and enhance left ventricular function [66,67,68]. Furthermore, HDAC1 is involved in the process of myocardial fibrosis by regulating the transcription factor GATA4. Previous studies have shown that the interaction between HDAC1 and histone acetyltransferase (GCN5) can modulate the activity of GATA4, thereby promoting the differentiation of mesenchymal stem cells into cardiomyocytes [69]. Another study revealed that the loss of GATA4 expression in endothelial cells exacerbates the fibrotic process by regulating platelet-derived growth factor B (PDGFB)-mediated pro-fibrotic vasocrine signaling pathways [18]. The results of this study indicate that protocatechuic acid treatment suppressed the upregulation of HDAC1 and the downregulation of GATA4 in perivascular cells and tissues of the myocardial fibrosis model rats, while reducing the mesenchymal marker α-SMA and promoting the expression of the endothelial junction protein CD31. This finding suggests that abnormal activation of the HDAC1/GATA4 signaling pathway may be a critical link connecting myocardial injury and fibrotic responses. In addition, MS-275 was used as an HDAC inhibitor in this study for mechanistic alignment. Although this compound is often considered a selective HDAC inhibitor, it does not target HDAC1 exclusively and retains activity against other class I HDAC subtypes, such as HDAC3 [70]. Thus, the observed pharmacological effects may partially involve contributions from isoforms, including HDAC3. Future research should employ more subtype-selective inhibitors or conduct endothelial cell-specific gene knockout experiments to more precisely delineate the distinct contributions of individual HDAC isoforms to the anti-fibrotic mechanism of protocatechuic acid.

Based on the above theoretical predictions, we conducted validation at the cellular level. During the Ang II-induced inflammatory response and EndMT in endothelial cells, the HDAC1/GATA4 signaling pathway was activated, accompanied by the activation of the NF-κB signaling pathway and increased release of inflammatory factors TNF-α, IL-6, and IL-1β. After intervention with protocatechuic acid, these pathological changes were significantly reversed, and the inflammatory response was effectively suppressed. Notably, specific knockdown of HDAC1 using siRNA successfully mimicked the protective effects of protocatechuic acid. However, combined intervention with protocatechuic acid and HDAC1 knockdown did not show statistically significant differences compared with either intervention alone, suggesting that the HDAC1/GATA4 signaling pathway likely represents the key molecular mechanism through which protocatechuic acid inhibits vascular endothelial inflammation and subsequent fibrotic process, thereby exerting its anti-myocardial fibrotic effects. These results suggest that the HDAC1/GATA4 signaling axis and the NF-κB pathway may collectively participate in the regulation of myocardial fibrosis, and their functions are closely related to the cardioprotective mechanism of protocatechuic acid.

To identify the potential molecular targets mediating the protective effects of protocatechuic acid, this study utilized molecular docking to analyze its interaction with a key protein, HDAC1. The result revealed a strong binding affinity between protocatechuic acid and HDAC1. Subsequently, we systematically evaluated the stability of the resulting protein-ligand complex through molecular dynamics simulations. A 100 ns simulation was performed to examine the binding stability at the HDAC1 active site. Key parameters, including RMSD, Rg, SASA, and RMSF, indicated that the complex remained relatively stable under physiological-like conditions. Finally, binding free energy calculations further confirmed the high affinity of protocatechuic acid for HDAC1. Collectively, these computational findings corroborate the earlier experimental results.

In contrast to synthetic HDAC inhibitors in clinical or preclinical development, protocatechuic acid exhibits a distinct dual character. Most approved or investigational synthetic HDAC inhibitors—such as vorinostat and MS-275 used in this study—are pan-HDAC or class I-HDAC inhibitors, whose severe dose-limiting toxicities considerably restrict long-term use in chronic conditions [71]. Our findings indicate that protocatechuic acid may show relative selectivity toward HDAC1, and this “selective inhibition” profile could offer a wider therapeutic window. However, it also presents clear translational hurdles, primarily concerning its potency, pharmacokinetics, and potential off-target effects during long-term administration. Therefore, protocatechuic acid should be positioned as a well-tolerated, long-term usable “modulator” in clinical translation. Future research and development should prioritize optimizing the drug targeting capability of protocatechuic acid and systematically completing its preclinical safety assessment.

The limitations of this study should be considered. First, although the ISO-induced model used is a classic approach for studying myocardial fibrosis, its acute/subacute pathological processes do not fully replicate the slow, progressive fibrotic evolution seen in human chronic cardiovascular diseases such as long-term hypertension or ischemic cardiomyopathy. This constrains the generalizability of our findings. Future studies should validate the efficacy of protocatechuic acid in more clinically relevant chronic models. Second, the HDAC1 inhibitor administered in vivo acts systemically and lacks cardiac endothelial-specific targeting. Furthermore, its inhibition of HDAC1 is not entirely specific, implying that the observed effects may involve multiple cell types and different HDAC subtypes. Third, although computational analyses suggest an interaction between protocatechuic acid and HDAC1, direct experimental validation is still lacking. Fourth, key pharmacokinetic parameters—such as oral bioavailability, tissue distribution, and half-life—as well as potential side effects on major organs following long-term administration were not addressed in this study. Fifth, the sample size in the experimental groups was relatively small. While statistically significant differences were observed across groups and these trends were consistent with histological and functional outcomes, the limited sample size may have reduced statistical power and constrained the precision of effect size estimation.

To address this gap and further delineate protocatechuic acid’s anti-fibrotic mechanisms, future research will focus on the following aspects. First, we will establish a cardiac endothelial-specific HDAC1 knockout mouse model. Second, experimental approaches such as surface plasmon resonance or competitive binding assays will be employed to verify the protocatechuic acid-HDAC1 interaction. Finally, we will systematically conduct preclinical pharmacokinetic and long-term safety evaluations of protocatechuic acid, expand the sample size, and test its efficacy in multiple animal and cell models of cardiac fibrosis. These studies are designed to observe its function in the aforementioned model and clarify its underlying mechanisms, which will be vital for providing deeper insights into protocatechuic acid’s protective role against myocardial fibrosis.

In summary, this study elucidates the mechanism through which protocatechuic acid attenuates myocardial fibrosis by modulating the HDAC1/GATA4 signaling pathway, thereby suppressing intravascular inflammation and regulating EndMT. Importantly, we uncover a previously overlooked protective mechanism wherein protocatechuic acid inhibits the progression of myocardial fibrosis by facilitating endothelial cell-mediated remodeling of the cardiac microenvironment. Mechanistically, protocatechuic acid promotes endothelial repair, reduces inflammatory injury and EndMT, and inhibits the release of pro-inflammatory cytokines (e.g., TNF-α, IL-6, IL-1β) as well as the activation of signaling pathways such as NF-κB. Consequently, it alleviates intravascular inflammation and disrupts the “inflammation-fibrosis” vicious cycle, a process critical for maintaining vascular integrity.

## 5. Conclusions

By demonstrating that protocatechuic acid alleviates myocardial fibrosis through suppression of inflammation and EndMT through the HDAC1/GATA4 signaling pathway, this study identifies a promising therapeutic candidate, thereby establishing a crucial foundation for developing novel anti-fibrotic strategies.

## Figures and Tables

**Table 1:** The list of antibodies used in this study.

Antibody Name	Product Code	Company Name
CD31	ER31219	HuaAn Biotechnology
α-SMA	ET1607-53	HuaAn Biotechnology
HDAC1	ET1605-35	HuaAn Biotechnology
GATA4	19530-1-AP	Proteintech
P-NF-κB p65(Ser536)	310013	Zen-Bioscience
NF-κB p65	80979-1-RR	Proteintech
TGFβ	HA721143	HuaAn Biotechnology
Collagen I	14695-1-AP	Proteintech
GAPDH	ET1601-4	HuaAn Biotechnology
Multi-rAb^®^ CoraLite^®^ Plus 594-Goat Anti-Rabbit Recombinant Secondary Antibody (H + L)	RGAR004	Proteintech
HRP Conjugated Goat anti-Rabbit IgG polyclonal Antibody	HA1001	HuaAn Biotechnology
HRP-conjugated Mouse anti-Rabbit IgG Light Chain	AS061	ABclonal

**Table 2:** Sequences of the primers for qRT-PCR.

Gene	Forward (5′-3′)	Reverse (5′-3′)
HDAC1	CGCCCTCACAAAGCCAATG	CTGCTTGCTGTACTCCGACA
GATA4	CGACACCCCAATCTCGATATG	GTTGCACAGATAGTGACCCGT
TNFα	ATGAGCACTGAAAGCATGATCCG	AGGAGAAGAGGCTGAGGAACAAG
IL-6	AGCCACTCACCTCTTCAGAACG	TGCCTCTTTGCTGCTTTCACAC
IL-1β	GCACCTGTACGATCACTGAACTG	CACTTGTTGCTCCATATCCTGTCC
GAPDH	ACCATCTTCCAGGAGCGAGA	GATGACCCTTTTGGCTCCCC

## Data Availability

The original contributions presented in this study are included in the article/Supplementary Material. Further inquiries can be directed to the corresponding author.

## Supplementary Materials

The following supporting information can be downloaded at: https://www.mdpi.com/article/10.3390/biology15020206/s1. File S1: Full-length, uncropped Western Blots.

## Abbreviations

The following abbreviations are used in this manuscript:

EndMT	endothelial-to-mesenchymal transition
ISO	isoproterenol hydrochloride
PCA	protocatechuic acid
HUVECs	human umbilical vein endothelial cells
Ang II	angiotensin II
ECM	extracellular matrix
HDAC1	histone deacetylase 1
NF-κB	nuclear factor-kappa B
GATA4	GATA Binding Protein 4
IL-6	interleukin-6
IL-1β	interleukin-1 beta
α-SMA	α-Smooth Muscle Actin
CD31	platelet Endothelial Cell Adhesion Molecule-1
H&E	hematoxylin and eosin
IACUC	institutional Animal Care and Use Committee
LVEF	left ventricular ejection fraction
LVFS	left ventricular fractional shortening
LVPWD	left ventricular posterior wall thickness at end-diastole
LVEDV	left ventricular end-diastolic volume
LVESV	left ventricular end-systolic volume
LDH	lactate dehydrogenase
CK-MB	creatine kinase isoenzyme-MB
NO	nitric oxide
CVF	collagen volume fraction
PDB	protein data bank
MM/PBSA	molecular mechanics/poisson-boltzmann surface area
FBS	fetal bovine serum
RMSD	root mean square deviation
Rg	radius of gyration
SASA	solvent-accessible surface area
RMSF	root mean square fluctuation
FEL	free energy landscape
LFE	lowest free energy
Co-IP	co-immunoprecipitation
RAAS	renin–angiotensin–aldosterone system
