# Supplementary figures and images for: Suppressing Endothelial–Mesenchymal Transition Through the Histone Deacetylase 1/GATA Binding Protein 4 Pathway: The Mechanism of Protocatechuic Acid Against Myocardial Fibrosis Revealed by an Integrated Study
Source: Biology (Basel). 2026 Jan 22;15(2):206. doi: 10.3390/biology15020206 (PMC12837532; doi:10.3390/biology15020206)

Figure3 (I)

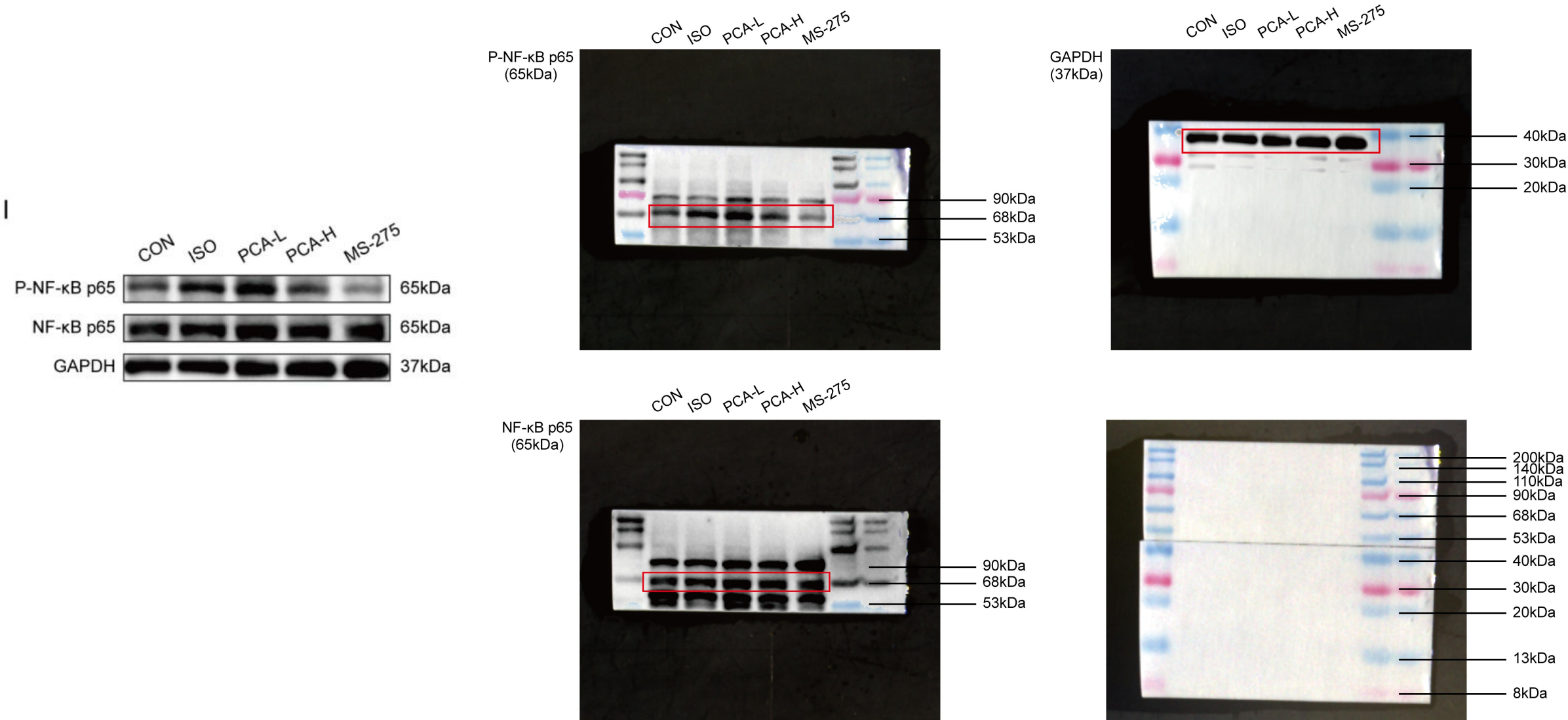

Figure4 (I)

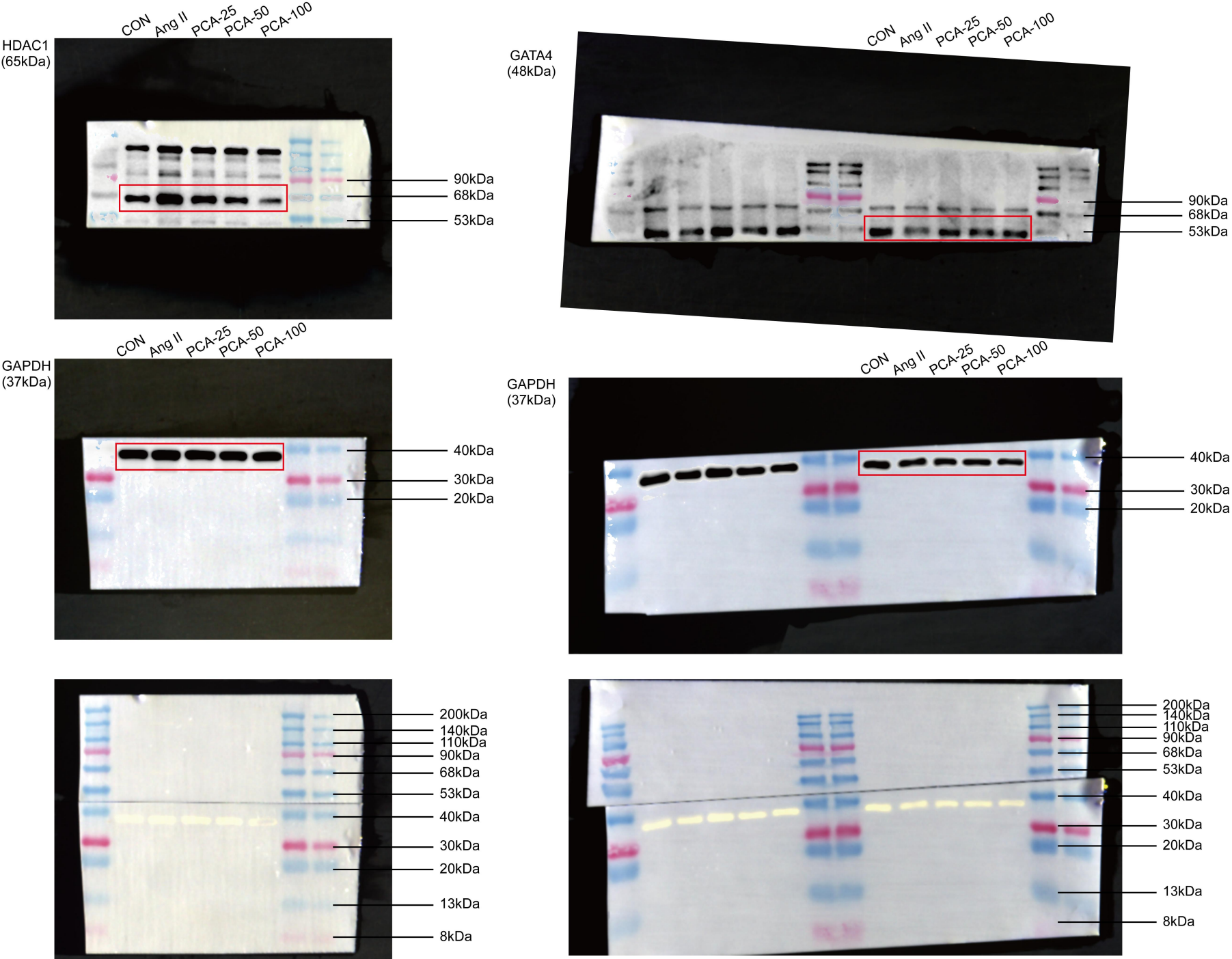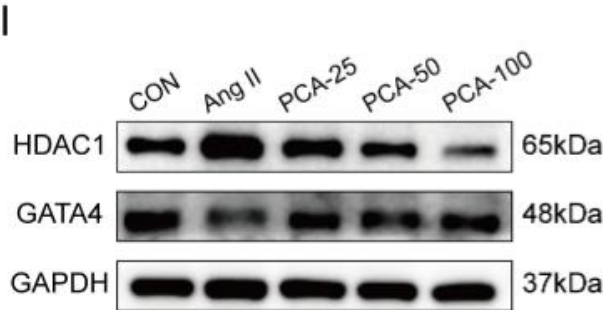

Figure4 (K)

K

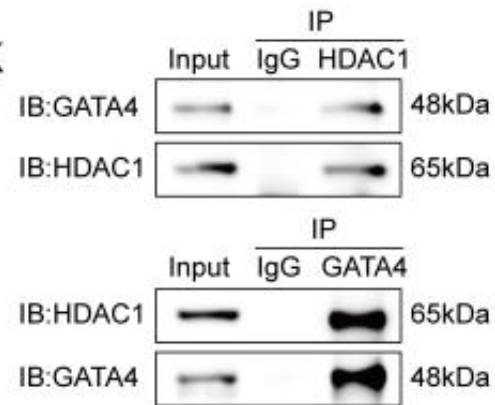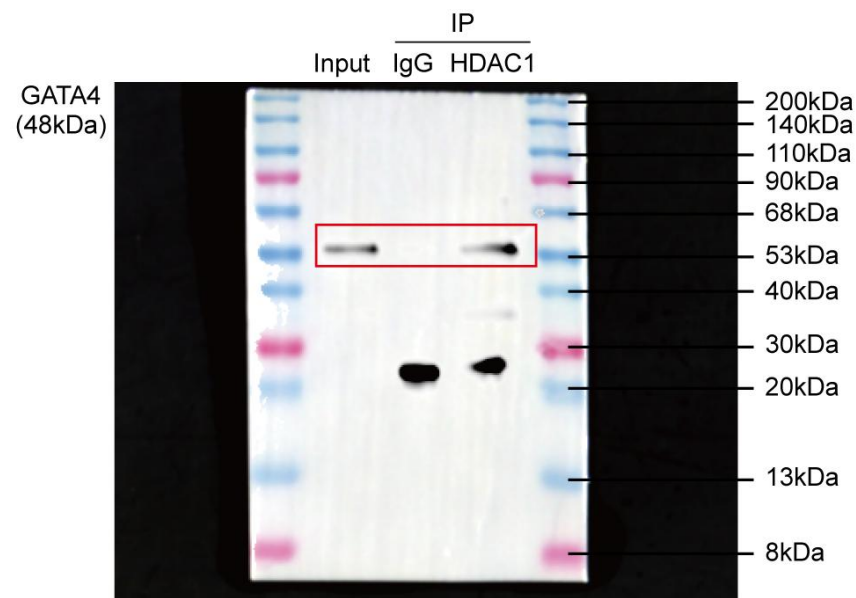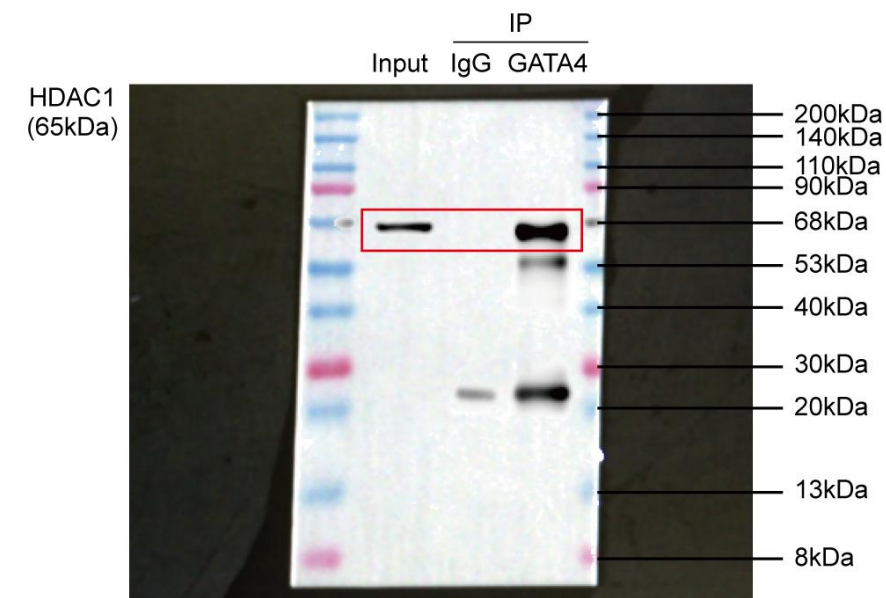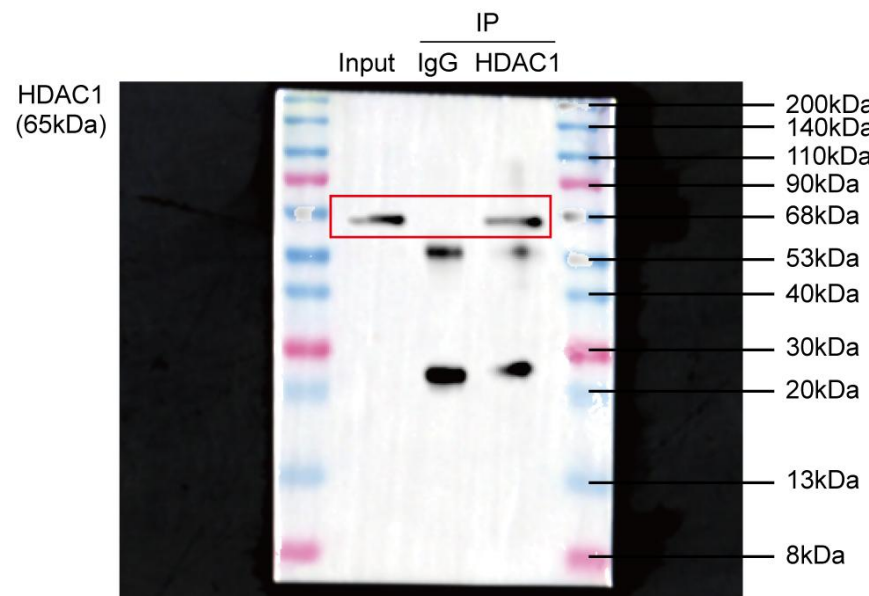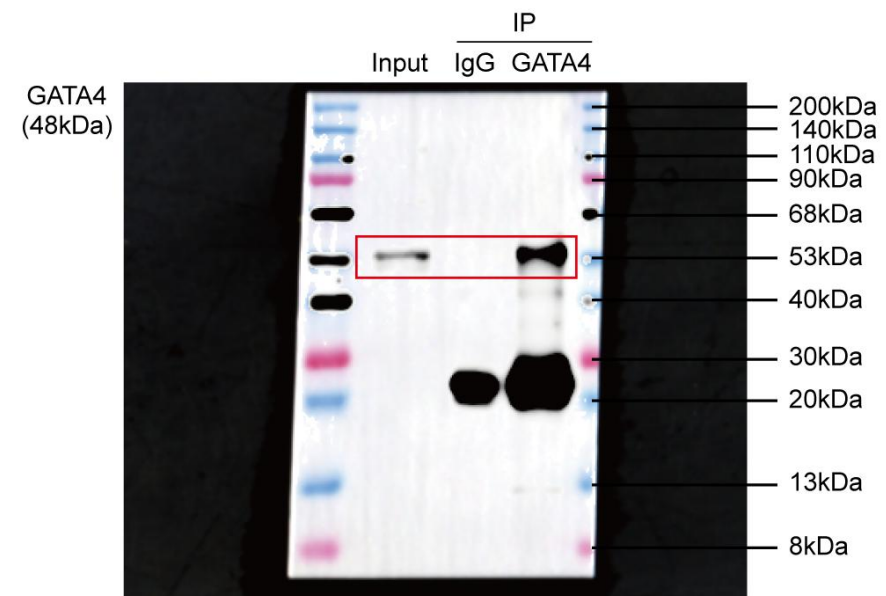

Figure5 (A)

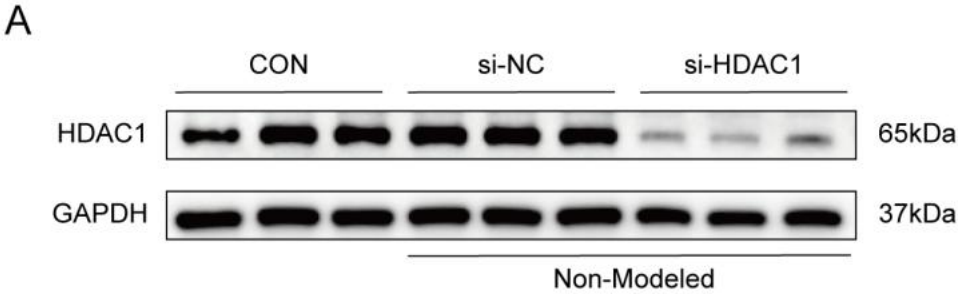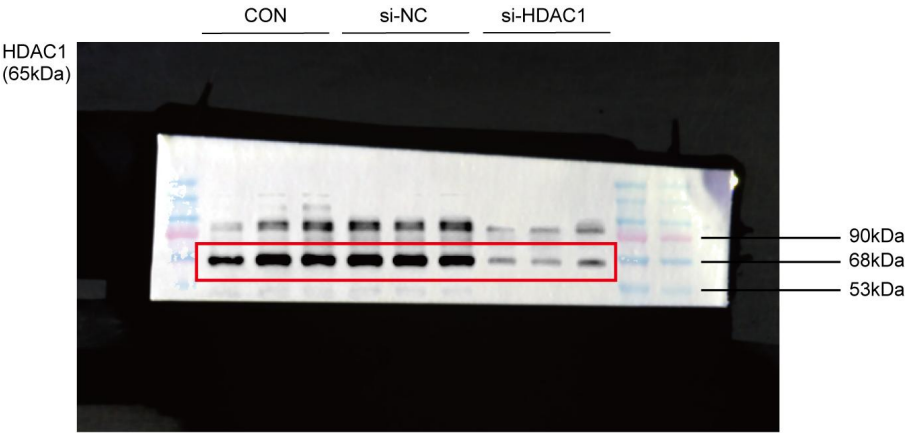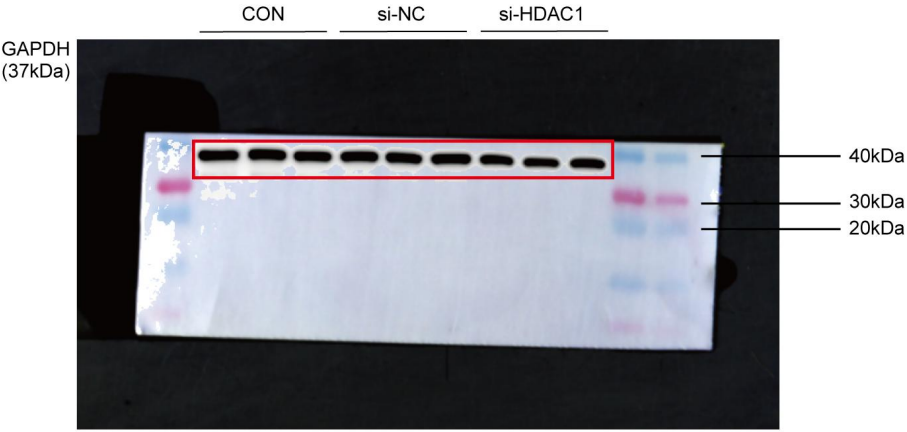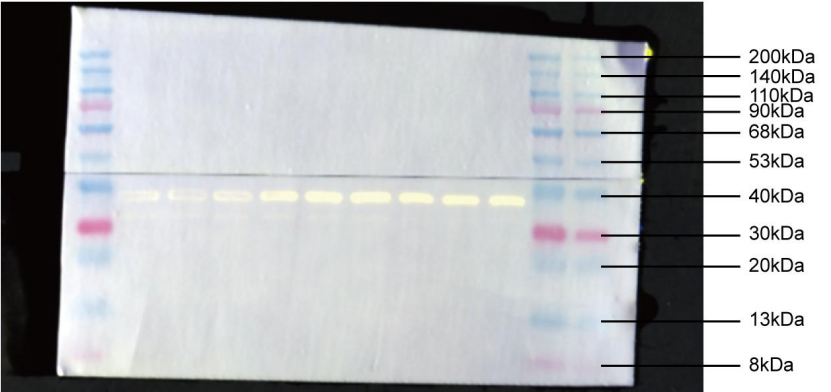

Figure5 (C) -GATA4

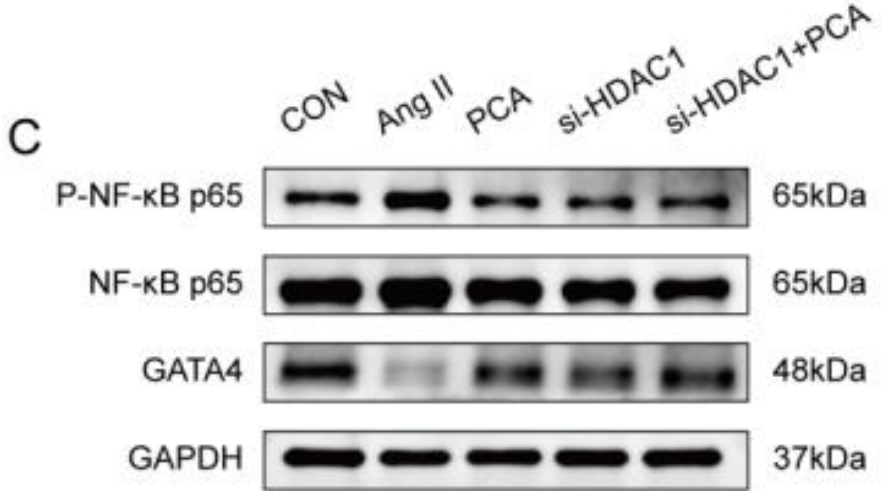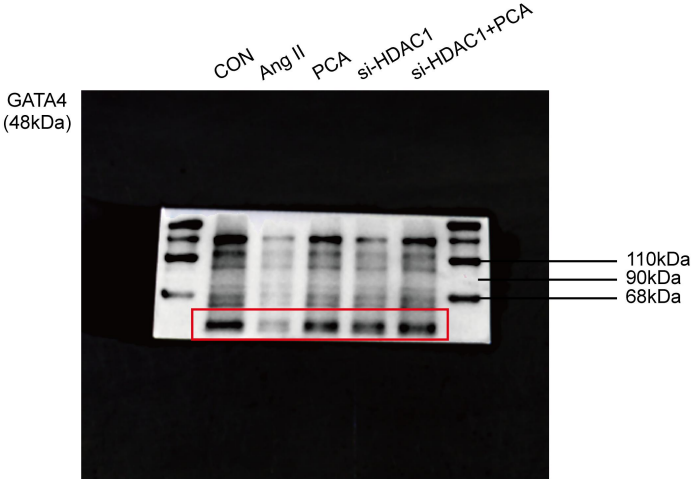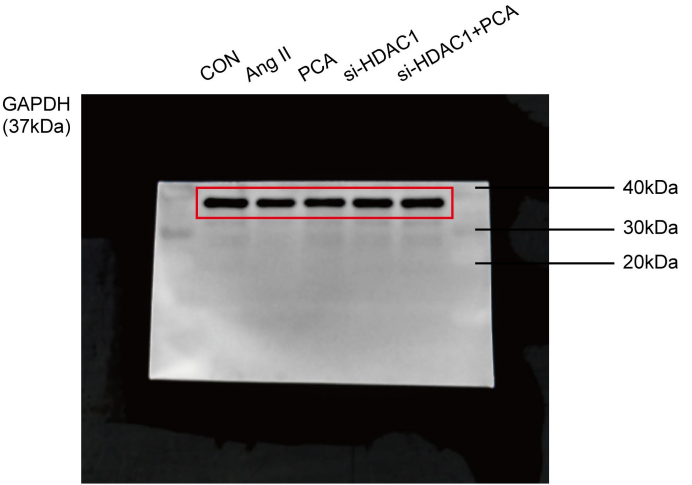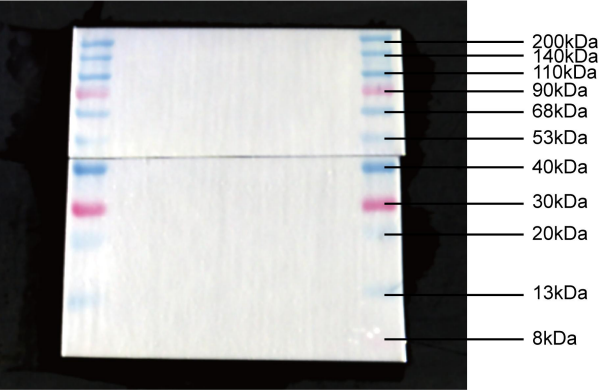

Figure5 (C) -P-NF-κB p65

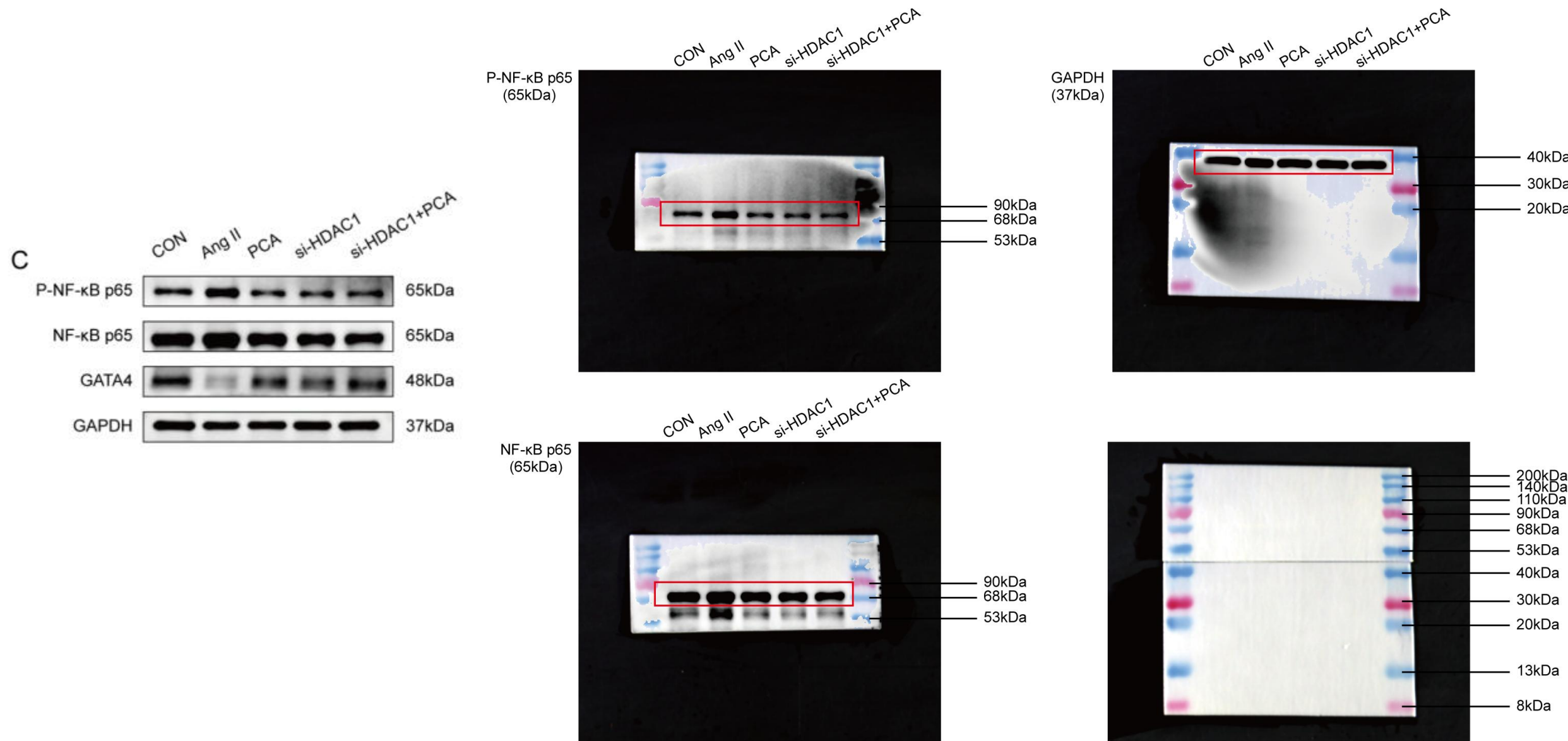

Figure5 (G)

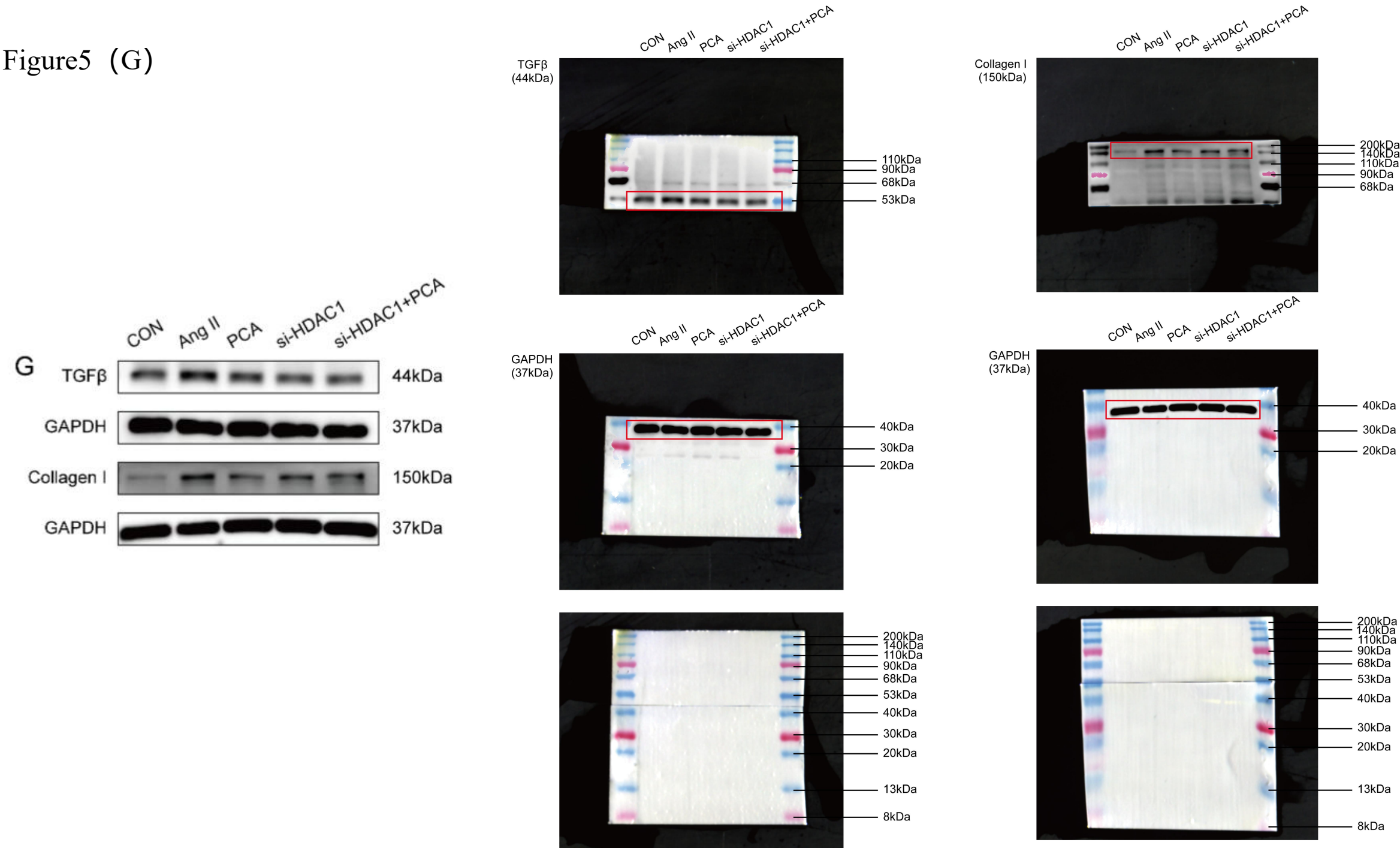

Supplement: Supplementary file 1 [file biology-15-00206-s001.zip › File S1 Full-length, uncropped Western Blots.pdf]
